# The psychometric properties of the Chinese version—reintegration to normal living index (C-RNLI) for identifying participation restriction among community-dwelling frail older people

**DOI:** 10.1186/s12877-017-0424-5

**Published:** 2017-01-31

**Authors:** Justina Yat-Wa Liu, Ka Wai Ma

**Affiliations:** Centre for Gerontological Nursing, School of Nursing, The Hong Kong Polytechnic University, Hung Hom, Hong Kong

**Keywords:** Frailty, WHO-ICF, Participation restriction, Psychometric properties

## Abstract

**Background:**

The Reintegration to Normal Living Index (RNLI) was developed to measure reintegration to normal living after major traumas/illnesses. Its psychometric properties remain unknown when used to measure participation restriction under the World Health Organization’s International Classification of Functioning, Disability, and Health (WHO-ICF) framework. This study examines the psychometric properties of the Chinese version-RNLI to measure WHO-ICF participation restriction among community-dwelling pre-frail and frail older people.

**Methods:**

A cross-sectional study was conducted in community and day-care centres in Hong Kong between May 2015 and January 2016. Through face-to-face interviews, information was collected on the participants’ demographic background, medical history, frailty status, depressive mood, functional performance in daily activities, and participation restriction. The internal consistency, test-retest reliability, and construct and convergent validity of the C-RNLI were assessed.

**Results:**

Two hundred and ninety-nine pre-frail or frail community-dwelling older people with a mean age of 79.53 were recruited. A confirmatory factor analysis showed that the C-RNLI has a two-factor structure comprised of “participation in physical activities” and “participation in social events”. The test-retest coefficient was 0.71. The Cronbach’s alpha of the total C-RNLI score, and those of the factors “participation in physical activities” and “participation in social events” were 0.88, 0.82 and 0.84, respectively. Pre-frail older people had significantly higher scores for the factors “participation in physical activities” (z = −5.05, <0.01) and “participation in social events” (z = −6.04, *p* < 0.01) than frail older people. Older people from community centres had significantly higher scores for the factors “participation in physical activities” (z = −4.48, <0.01) and “participation in social events” (z = −4.03, *p* < 0.01) than older people from day-care centres. The factors “participation in physical activities” and “participation in social events” of the C-RNLI were significantly convergent with depressive mood (r_s_ = −0.25 and r_s_ = −0.39, respectively) and functional performance in daily activities (r_s_ = 0.28 and r_s_ = 0.45, respectively).

**Conclusions:**

The C-RNLI is a two-factor structured scale with acceptable level of reliability and validity to measure WHO-ICF participation restriction among community-dwelling pre-frail and frail older people.

## Background

The International Classification of Functioning, Disability, and Health (WHO-ICF) model offers a framework for classifying and understanding the influences on people’s physio-psycho-social health and how disability can impact the daily life of sufferers on three levels: through impaired body functions or structures, activity limitations, and participation restriction [1]. Participation restriction refers to how the disability is limiting a sufferer’s involvement in valued life events [1]. The nine domains of WHO-ICF participation restriction include learning and applying knowledge, general tasks and demands, communication, mobility, self-care, domestic life, interpersonal interactions and relationships, major life areas and community, and social and civic life [1]. About 51.8% of community-dwelling older adults reported having difficulties engaging in at least one WHO-ICF domain of participation [2]. The ability to be involved in various life events was found to be more important than physical health for older people to achieve successful ageing [3]. Participation restriction is strongly associated with many health problems such as pain, anxiety, depression, cognitive impairment, and disability [4], eventually leading to early institutionalization and social isolation for older people.

Reintegration to normal living (RNL) is about the ability of an individual to reform his/her physio-psycho-social characteristics into a harmonious whole so as to resume living a well-adjusted life after an incapacitating illness or trauma [5]. The Reintegration to Normal Living Index (RNLI) is an 11-item scale that was validated to measure RNL. The RNLI has been translated and validated in many studies involving patients from hospitals and rehabilitation centres [6, 7] with mobility limitations [8], spinal cord injuries [9, 10], and stroke [11, 12]. Although the concept of RNL is different from that of WHO-ICF participation restriction, the RNLI has been used to measure levels of participation restriction in stroke patients [13, 14], wheelchair users [15], and frail older people [16].

Frailty refers to a physiological state of increased vulnerability to stressors resulting from a decrease and possible dysregulation of reserves in multiple physiological and/or biological systems. Frailty can be viewed as a process of deterioration in physical functions. Some older people may experience pre-frailty before becoming frail [17, 18]. Frailty, disability, and comorbidity are in fact separate clinical states that require distinct prevention and therapeutic strategies. The early stages of frailty may be clinically silent, with 32.3% of frail older people having neither disabilities nor comorbidities [18]. Although the RNLI has been recommended for use in measuring participation restriction among people with different illnesses [13, 16, 19], the psychometric properties of the RNLI for measuring WHO-ICF participation restriction among community-dwelling older people with pre-frailty/frailty remain unknown. It is important to have a validated instrument to assess the levels of WHO-ICF participation restriction of older people in stages of frailty (i.e., pre-frailty and frailty). Such an instrument will help in the effort to identify the problem in its early stages according to frailty levels and to evaluate the effectiveness of any interventions, so as to be able to address problems related to participation restriction among this special group of older people. Therefore, the aim of this study was to establish the psychometric properties of the C-RNLI, including its internal consistency, test-retest reliability, and construct validity (including hypothesis-testing based convergent validity and factor-based validity) when measuring participation restriction among pre-frail and frail community-dwelling older people.

## Methods

### Settings and participants

Two hundred and ninety-nine participants were recruited from five community elderly centres and nine day-care centres for the elderly in Hong Kong between May 2015 and January 2016. A convenience and snowball sampling method was used to recruit community-dwelling older people aged ≥ 65 who could communicate in Cantonese and who had been assessed as being in a pre-frail or frail state according to the Fried Frailty Index (FFI) [20]. The items in the index included: i) unintentional weight loss: a self-reported unintentional loss of 10% of body weight in the past year; ii) exhaustion: by answering “Yes” to either “I felt that everything I did was an effort” or “I could not get going in the last week”; iii) slowness: a 4.5-m walk with an average walking speed in the lowest quintile stratified by median body height; iv) weakness: with a maximal grip strength, as measured by hand dynamometers, in the lowest quintile stratified by the body mass index quartile; and v) low activity: a Physical Activity Scale for the Elderly-Chinese (PASE-C) score in the lowest quintile (i.e., < 30 for men and < 27.5 for women). The presence of ≧ 3 items indicated that the elderly person was in a state of frailty, while 1–2 items indicated pre-frailty [20].

Participants were excluded if they were cognitively impaired (with an abbreviated mental test score of < 6), had been admitted to hospital in the past 6 months, or were confined to bed or restricted by the permanent use of a wheelchair.

### Measurement

Participation restriction was measured by the C-RNLI, which was translated from the RNLI. The translation process followed standard procedures involving translation and back-translation [21]. A professional translator first produced a provisional translated version of the RNLI. This provisional version was then back-translated by LJWY (the first author). A comparison was made between the back-translated version and the RNLI, and the discrepancies between them were discussed. The required modifications were made and a pre-final version of the C-RNLI was drawn up. Then, five older people were invited to comment on the pre-final version of the C-RNLI in terms of its difficulty, quality, clarity, and language use. The pre-final version of the C-RNLI was reviewed by the research team according to the feedback from the older people, and the final C-RNLI was produced.

Although Pang et al. [9] had developed the Chinese version of the RNLI based on the extent to which 75 patients with chronic stroke had reintegrated to normal living [9], in this study it was decided that a new version of the C-RNLI should be developed. This was because the target participants of this study were frail older people, most of whom had received little or no education when they were young because of the disruption of the war years, and hence had a low level of literacy [22]. Thus, a version of the C-RNLI using simple words and structures was needed to ensure that they would be able to understand it. This was achieved by soliciting the opinions of a gerontologist and a group of frail older people during the process of developing the C-RNLI in this study. The C-RNLI consists of 11 declarative statements rated on an 11-point numerical rating scale (with 0 indicating the least agreement and 10 the greatest agreement with the statements). Item scores were summed and proportionally converted to 100 through dividing the score by 1.1 to provide a total score, with a lower score indicating a higher level of participation restriction [5].

The original RNLI was validated among 109 patients from hospitals and rehabilitation centres [4, 5]. The Cronbach’s alpha of the RNLI ranged from 0.87 to 0.97 in studies involving patients with stroke [11], spinal cord injuries [8], and limited mobility [7], which supported its internal consistency with a measure construct of RNL. The two-factor structure of the RNLI was first proposed by Wood-Dauphinee et al. [5]. The scores from items 1 to 8 were summed to give the total score of factor 1, which was called “daily functioning”; and the scores from items 9 to 11 were summed to give the total score of factor 2, which was called “perception of self” [5]. This factor structure was also found in the Chinese version of the RNLI developed by Pang et al. [9]. However, the exploratory factor analysis conducted by Stark et al. [7] showed another two-factor structure for the RNLI when the RNLI was validated with community-dwelling people with limited mobility [7] and comorbidities [6]. For this factor structure, the scores from items 1 to 5 were summed to give the total score of factor 1, which was called “physical reintegration”; and the scores from items 6 to 11 were summed to give the total of factor 2, which was called “social reintegration”.

The depressive mood of the participants was measured by the Cantonese version of the Geriatric Depression Scale Short Form (CGDS-SF) [23]. The participants were required to answer yes or no to 15 statements describing different emotions. The CGDS-SF had good internal consistency, test-retest reliability, criterion-related validity [24], and good sensitivity and specificity for identifying geriatric depression [25]. The scores of the items were summed to provide the total CGDS-SF score, where a higher score indicated a higher level of depressive mood. Under the WHO-ICF, depressive mood is a kind of impaired body function [1], and it has been found to be associated with participation restriction [16]. Therefore, it was hypothesized in this study that a negative correlation would be found between the C-RNLI and the CGDS-SF to establish the convergent validity of the C-RNLI in relation to participation restriction.

The functional performance in daily activities of all of the participants was measured using the Hong Kong Chinese version of the 9-item Lawton Instrumental Activities of Daily Living Scale (HKC-IADL) [26]. Each item was rated on a 4-point Likert scale. All item scores were summed to provide the total score, with a lower score indicating a higher level of dependence. The scale showed good internal consistency, test-retest reliability, inter-rater reliability, and construct validity [27]. The HKC-IADL was used to measure activity limitations, which is a dysfunction level of the WHO-ICF [1], and it was found that older people who frequently participate in social activities were less likely to display disabilities in the instrumental ADLs [28]. Thus, it was hypothesized in this study that a positive correlation would be found between the C-RNLI and the HKC-IADL to establish the convergent validity of the C-RNLI in relation to participation restriction.

### Procedures

Flyers introducing the aims of the study were posted at the community and day-care centres and staff at those centres also promoted this study verbally to their members in regular meetings. Through the centres, interested older people signed up to participate. The eligibility of the participants to take part in this study was assessed by the research assistants (RAs) according to the selection criteria. Ethical approval for the study was obtained from the Human Subject Ethics Committee of the Hong Kong Polytechnic University. Written informed consent was obtained from all of the participants before data were collected. A group of RAs used a structured questionnaire to collect demographic and other measurements from the participants. To establish the test-retest reliability of the C-RNLI, 1 month after the first batch of data were collected, thirty participants were randomly selected and assigned to one RA out of the same group of RAs to have their level of participation restriction evaluated again.

### Data analysis

The data were analysed using the Statistical Package for the Social Sciences 21.0 (SPSS Inc., Chicago, IL), and the confirmatory factor analysis (CFA) was conducted using the Stata Statistical Software 14 (StataCorp., Texas, TX). Descriptive statistics were used to evaluate the demographic data of the participants as well as the other assessments. The reliability of the scale was established using internal consistency and test-retest reliability. Cronbach’s alpha was used to measure the internal consistency of the scale, with a reliable scale having a Cronbach’s alpha of > 0.7 [29]. Intra-class coefficient (ICC) (2,1) was used to measure test-retest reliability, with an ICC of > 0.75 indicating good reliability and an ICC of between 0.5 and 0.75 indicating moderate reliability [30]. The item-total correlation and the Cronbach’s alpha were also checked after items were deleted. A reliable item should have an item-total correlation of > 0.3 [31] and should not cause the Cronbach’s alpha to become larger after it is deleted. A Spearman’s rank correlation coefficient (r_s_) was used to establish hypothesis-testing based construct validity, with a correlation coefficient of between 0.1 and 0.29 considered a small effect, that between 0.3 and 0.49 considered a moderate effect, and that ≧0.5 considered a large effect [32]. It was expected that the C-RNLI would be significantly correlated with the CGDS-SF negatively and with the HKC-IADL positively.

The known-groups method was used to establish construct validity. All of the participants were classified based on their frailty status and whether they had been recruited from community versus day-care centres. The participants from the day-care centres were generally more impaired in their ability to maintain their optimal level of daily activities, and less capable of taking care of themselves than the participants from the community centres. It was hypothesized that participants recruited from day-care centres would have significantly lower C-RNLI scores than participants from community centres. Likewise, participation restriction was found to be more prevalent in frail older people [16]. Therefore, it was hypothesized that frail participants would have significantly lower C-RNLI scores than pre-frail participants. A Mann-Whitney *U* test was used to determine whether there were significant differences between these two groups in C-RNLI scores. A value of *p* < 0.05 was considered statistically significant.

The CFA was used to establish the structural validity of the C-RNLI. A CFA with a maximum likelihood estimation was conducted for the respective factor structures proposed by Wood-Dauphinee et al. [5] and Stark et al. [7] to identify which one would be the most appropriate factor structure for the C-RNLI in this study. An acceptable model fit would be indicated by: i) $\frac{\mathrm{chi}\hbox{-} \mathrm{squared}}{\mathrm{degree}\;\mathrm{of}\;\mathrm{freedom}}\le 3$; ii) a root mean square error of approximation (RMSEA) score of ≤ 0.08, iii) a comparative fit index (CFI) score of ≥ 0.95; and vi) a Tucker-Lewis Index (TLI) score of ≥ 0.90 [33–37].

## Results

The mean age of the participants was 79.53 (SD 7.33), their mean C-RNLI score was 68.31 (SD 19.64), 223 (74.58%) of the participants were female, 151 (50.50%) were widowed, 157 (52.51%) had received a primary education, and 139 (46.49%) were identified as frail according to the FFI (Table 1) [20].

**Table 1:** Descriptive statistics for the demographic, physiological, and psychological measures (*N* = 299)

Characteristics		*Number*	Percent
Demographic variables			
Gender	Male	76	25.42
	Female	223	74.58
Centre	Day-care	95	31.77
	Community	204	68.22
Marital status	Not married	13	4.35
	Married	121	40.47
	Widowed	151	50.50
	Divorced/Separated	13	4.35
	Missing	1	0.33
Education level	No formal education	90	30.10
	Primary level	157	52.51
	Secondary level	43	14.38
	Degree level	6	2.01
	Others	1	0.33
	Missing	2	0.67
Fried Frailty Index	Pre-frail	160	53.51
	Frail	139	46.49
Weight loss	Yes	12	4.01
Slowness	Yes	140	46.82
Weakness	Yes	244	81.61
Low activity	Yes	53	26.63
Exhaustion	Yes	299	100
Characteristics		Mean	SD
Age		79.53	7.33
CGDS-SF		4.36	3.58
HKC-IADL		20.37	6.15
C-RNLI		68.31	19.64
C-RNLI–participation in physical activities		31.95	7.96
C-RNLI–participation in social events		43.19	15.66

*Abbreviations*: *CGDS-SF* Cantonese version of the Geriatric Depression Scale Short Form, *HKC-IADL* Hong Kong Chinese version of the Lawton instrumental activities of daily living scale, *C-RNLI* Chinese version of the Reintegration to Normal Living Index

### Construct validity–structural validity

There were no missing values in the responses to the C-RNLI items, so all of the data were used in establishing construct validity. The indicators of model fit for all four proposed models are presented in Table 2. Model 1 is a two-factor structure of the C-RNLI that was first proposed by Wood-Dauphinee et al. [5] and model 2 is another two-factor structure that was first proposed by Stark et al. [7]. Since none of the indicators of model fit reached an acceptable level for both models 1 and 2, these models were modified. All of the modifications were made according to each model’s modification index and the contents of the C-RNLI items. Model 1R was the revised model based on model 1. Paths of covariance between error terms were added according to the modification index of model 1. However, those indicators of model 1R still did not reach acceptable levels.

**Table 2:** Indicators of model fit: Indices of the CFA of the four proposed models of the C-RNLI

Model	Factor	C-RNLI items	Chi-squared (chi-squared / df)	*p*-value	RMSEA (90% Confidence Interval)	CFI	TWI
1	Daily functioning	1–8	273.24 (6.35)	*p* = 0.00	0.13 (0.12, 0.15)	0.85	0.81
	Perception of self	9–11					
2	Physical reintegration	1–5	335.19 (7.80)	*p* = 0.00	0.15 (0.14, 0.17)	0.81	0.76
	Social reintegration	6–11					
1R	Daily functioning	1–8	181.36 (4.53)	*p* = 0.00	0.11 (0.09, 0.13)	0.91	0.88
	Perception of self	9–11					
2R	Participation in physical activities	1,2,4,5	115.94 (2.97)	*p* = 0.00	0.08 (0.06, 0.10)	0.95	0.93
	Participation in social events	3 & 6–11					

Criteria for acceptable models: i) a small chi-square, which has a non-significant value of *p* ≥ 0.05 or (chi-squared)/(degree of freedom) ≤ 3; ii) RMSEA ≤ 0.08, iii) CFI ≥ 0.95; and vi) TLI ≥ 0.90 [33–37]

**Fig. 1:**
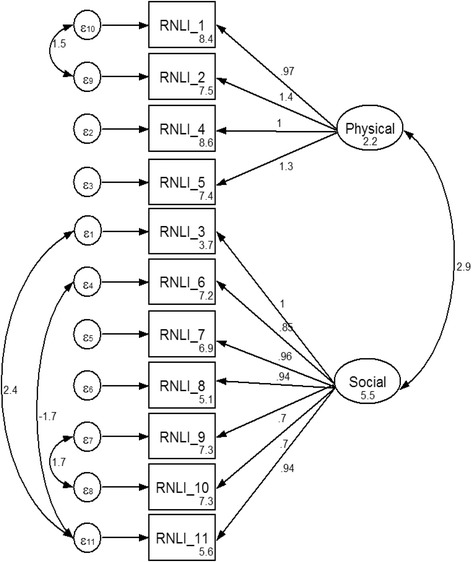
The model 2R of the CFA of the C-RNLI

Model 2R (Fig. 1) was the revised model based on model 2. Two factors of the C-RNLI were named “participation in physical activities” and “participation in social events” to match the measure construct of this new C-RNLI. Item 3 measures the extent to which older people are restricted in their ability to go on trips. It is suggested that taking a trip could be a stressful event [38], requiring older people to make social readjustments due to the stress of being separated from friends and family members during the trip. Moreover, social factors such as visiting family members/friends, travelling with family members/friends, and meeting and socializing with new people are important incentives for older people to go on a trip [39]. Therefore, it has been suggested that going on a trip is very much a socially based activity, so it was decided to put item 3 under the factor of “participation in social events” instead of under “participation in physical activities” in model 2R. Since both taking trips and engaging in recreational activities were common events in the lives of older people [38, 40], paths of covariance between the error terms of items 3 and 11 as well as items 6 and 11 were added. The path of covariance between the error terms of items 1 and 2 was added because both items measured the same WHO-ICF domain (i.e., ICF d460: Moving around in different locations) [1, 19]. The path of covariance between the error terms of items 9 and 10 was added because both items measured satisfaction with interpersonal relationships [19]. All of the newly added paths and changed paths were also suggested by the modification indices. All factor loadings of the items of the C-RNLI were significant in model 2R with *p* < 0.01. Factor loadings ranged from 0.97 to 1.40 in the factor “participation in physical activities” and from 0.70 to 1.00 in the factor “participation in social events”. All indicators of model fit for model 2R reached acceptable levels [33–37].

### Construct validity–hypothesis-testing based validity

The C-RNLI significantly correlated with CGDS-SF, with r_s_ = −0.38 (*p* < 0.01); and with HKC-IADL, with r_s_ = 0.44 (*p* < 0.01). The factor “participation in physical activities” of the C-RNLI was correlated with CGDS-SF), with r_s_ = −0.25 (*p* < 0.01); and with HKC-IADL, with r_s_ = 0.28 (*p* < 0.01). The factor “participation in social events” of the C-RNLI was correlated with CGDS-SF), with r_s_ = −0.39 (*p* < 0.01); and with HKC-IADL, with r_s_ = 0.45 (*p* < 0.01). Participation restriction is associated with various factors [2], and depression and activity limitation were found to be moderately associated with participation restriction [16, 28]. Therefore, the magnitudes of the correlation coefficients, which ranged from 0.25 to 0.45, were consistent with our prediction that the C-RNLI was moderately correlated with CGDS-SF and HKC-IADL.

The descriptive statistics of the C-RNLI and the differences in the C-RNLI scores between participants of different frailty status and centre type are presented in Table 3. The pre-frail group had a significantly higher C-RNLI score (z = −6.65, *p* < 0.01), “participation in physical activities” factor score (z = −5.05, <0.01), and “participation in social events” factor score (z = −6.04, *p* < 0.01) than the frail group. Participants from community centres had a significantly higher C-RNLI score (z = −4.60, *p* < 0.01), “participation in physical activities” factor score (z = −4.48, <0.01), and “participation in social events” factor score (z = −4.03, *p* < 0.01) than participants from the day-care centres.

**Table 3:** Descriptive statistics of the C-RNLI of participants of different frailty status and centre type

Characteristic	Group	Mean	SD	z-score	*p*-value
C-RNLI	Pre-frail group	75.12	18.41	−6.65	*p = 0.00
	Frail group	60.21	20.80		
	Community centres	72.08	17.91	−4.60	*p = 0.00
	Day-care centres	60.21	20.80		
C-RNLI-participation in physical activities	Pre-frail group	34.12	6.91	−5.05	*p = 0.00
	Frail group	29.46	8.37		
	Community centres	33.53	6.78	−4.48	*p = 0.00
	Day-care centres	28.56	9.18		
C-RNLI-participation in social events	Pre-frail group	48.51	15.00	−6.04	*p = 0.00
	Frail group	37.06	14.11		
	Community centres	45.75	14.95	−4.03	*p = 0.00
	Day-care centres	37.67	15.80		

*Abbreviations*: *C-RNLI* Chinese version of the Reintegration to Normal Living Index

*p < 0.05

### Reliability

The Cronbach’s alphas of the entire C-RNLI, the factor “participation in physical activities” and the factor “participation in social events” were 0.88, 0.82, and 0.84 respectively, which indicated that all of the scale items were consistent [30]. No Cronbach’s alpha became larger when any one item was deleted, and the corrected item-total correlations of the C-RNLI items ranged from 0.50 to 0.68 (Table 4), which indicated good item reliability [31]. The ICC (2,1) was 0.71, which indicated a moderate level of test-retest reliability [30].

**Table 4:** The original version of the RNLI and descriptive statistics for the items in the C-RNLI (*N* = 299)

Item in the Reintegration to Normal Living Index	Mean	SD	Corrected item-total correlation	Cronbach’s α after deletion
Q1. I move around my living quarters as I feel necessary. 我能在我的住處隨意走動。	8.44	2.18	0.59	0.87
Q2. I move around my community as I feel necessary. 我能在我的社區隨意走動。	7.48	2.97	0.62	0.87
Q3. I am able to take trips out of town as I feel are necessary. 有需要時,我可隨意安排行程出國。	3.65	3.88	0.50	0.88
Q4. I am comfortable with how my self-care needs (dressing, feeding, toileting, bathing) are met. 我能夠妥善照顧個人護理需求(穿衣,進食,如廁,洗澡) 感到滿意	8.59	2.11	0.56	0.87
Q5. I spend most of my days occupied in a work activity that is necessary or important to me. 我的精神體力足夠應付對我有必要或重要的活動上。	7.44	2.56	0.63	0.87
Q6. I am able to participate in recreational activities (hobbies, crafts, sports, reading, television, games, computers, etc.) as I want to. 我可以參與各類休閒活動 (例如:興趣,手工藝,運動,閱讀,電視遊戲,電腦等)。	7.24	2.83	0.61	0.87
Q7. I participate in social activities with family, friends, and/or business acquaintances as is necessary or desirable to me. 當有需要或對我適合時,我會參與親友及/或工作友好的社交活動。	6.94	3.16	0.66	0.86
Q8. I assume a role in my family that meets my needs and those of other family members. 在家庭的角色,我可以照顧家人的需求。	5.12	3.73	0.56	0.87
Q.9 In general, I am comfortable with my personal relationships. 總括而言,我很滿意我的人際關係。	7.32	2.35	0.68	0.86
Q.10 In general, I am comfortable with myself when I am in the company of others. 總括而言,我很滿意自已和別人相處時的表現。	7.32	2.32	0.67	0.86
Q.11 I feel that I can deal with life events as they happen. 我認為我可以處理不同人生的大事。	5.59	3.44	0.59	0.87

## Discussion

### Acceptable levels of reliability and validity for the C-RNLI

The results of the study indicated satisfactory item reliability and internal consistency, but only moderate test-retest reliability. According to the WHO-ICF model, participation restriction is influenced by impaired body functions, limitations on activities, and environmental, personal, and health factors [1]. Some participants might have received some treatments to improve their physical and psychological functioning during the study period. Therefore, the participants’ level of participation restriction might have changed due to those treatments, and 1 month might be too long to establish the test-retest reliability of the C-RNLI.

Both factors of the C-RNLI were significantly negatively correlated with depressive mood and positively correlated with the performance of IADLs among participants of different frailty status. These further support the previous findings of the relationship between participation restriction and these two common situations among frail older people [16, 28]. The results are also consistent with the view incorporated in the WHO-ICF model of a close association among three levels of dysfunction, namely impaired body functions (depressive mood), activity limitations (dependence in the IADL), and participation restriction [1, 16, 28]. Also, the C-RNLI was shown to be able to differentiate between older people with different levels of participant restriction due to various levels of frailty [16] and requiring various levels of care. These indicate that the C-RNLI has an acceptable level of hypothesis-based construct validity.

### Factor structure of the C-RNLI when measuring the WHO-ICF’s participation restriction

The CFA showed that the C-RNLI had a two-factor structure. Items 1–2 and 4–5 were under the factor called “participation in physical activities”, which was characterized by mobility (items 1–2) and ADLs (items 4 and 5). Items 3 and 6–11 were under the factor called “participation in social events”, which was characterized by social activities and life events (items 3, 7–8, and 11) and interpersonal relationships (items 9–10). In general, the results of this study support a two-factor structure such as the one proposed by Miller et al. [6] and Stark et al. [7], with the only discrepancy being whether item 4 should belong to the factor “participation in physical activities” or factor “participation in social events”. It was suggested that taking trips could be a social activity because social factors play an important role in taking trips and taking trips also requires social readjustment [38, 39]. Therefore, in this study it was reasonable to place item 4 under the factor “participation in social events”. On the other hand, a different factor structure of the C-RNLI was found from the factor structures proposed by Pang et al. [9] and Wood-Dauphinee et al. [5]. The differences in the factor structures may be due to the differences between the target populations (older people with frailty versus people with major illness) and the scoring systems (11-point scale vs 4 point scale) [7, 9]. Age might also account for the differences in factor structure. The mean age of the participants in this study was 79.54, making them much older than participants in other studies [5, 8–10]. It has been suggested that older people have decreased functional and cognitive abilities; therefore, the extent to which the participants would take part in activities would be affected by their age, especially in the case of social activities (items 5–8) [6, 41].

### Using the C-RNLI to assess WHO-ICF participation restriction among frail older people

Good reliability and validity were found in this study when all eleven C-RNLI items were used to measure participation restriction among older people with frailty. This is different from the suggestion that items 10 and 11 of the RNLI are unrelated to WHO-ICF participation restriction and should be deleted [19]. Examining the contents of items 10 and 11, it is clear that item 10 measures satisfaction with interpersonal interactions and item 11 measures the problem-solving skills that are employed when dealing with life events. Both interpersonal interactions and problem-solving skills are important elements in WHO-ICF participation restriction [1]. Therefore, there was no need to discard any items when using the C-RNLI to measure WHO-ICF participation restriction.

Previous studies to validate the RNLI mostly focused on populations with disabilities caused by major illnesses [5–10]. In those studies, the participants’ activities were initially greatly limited due to their impaired body functioning, and the RNLI was regarded as a tool to track the progress of the participants in recovering from their disability. However, frailty refers to a state in which older people are more vulnerable to negative health outcomes such as disability, dependency, or mortality [18]. People in the early stage of frailty could be clinically silent [18], but at risk of developing a disability that could affect their ability to take part in daily and social activities if sufficient attention is not paid to preventing this situation from happening. This study showed that the C-RNLI is a reliable and valid tool for detecting participation restriction among frail older people.

### Contrast between the C-RNLI and life space assessment

Comparing the C-RNLI and life space assessment, the items in the C-RNLI assess older people’s self-perceived levels of involvement and confidence in both physical and social events, whereas life space assessment requires people to report their habits of mobility in five life spaces (from the home to out of town) [40]. Life space assessment has shown a good correlation with RNLI [42]. As these two kinds of assessments differ to a certain extent, it is suggested that the C-RNLI be used together with the life space assessment to screen participation restriction in both physical and social events, to build up a more comprehensive and detailed understanding of the mobility-associated participation restriction of older people.

### Future directions for investigating psychometric properties of the C-RNLI

Although this study suggested that the C-RNLI has acceptable internal consistency, test-retest reliability, structural validity, and hypothesis-testing based construct validity, it is important to conduct a longitudinal study to ensure that the psychometric properties of the C-RNLI are stable over time. In addition, the participation levels of older people are likely to change over time due to deterioration in their body functions and the contracting of various diseases; therefore, the responsiveness of the C-RNLI should be investigated to ensure that the C-RNLI is a sensitive tool for monitoring the participation levels of older people. Furthermore, Fairhall et al. [16] recommended a cut-off point for using the RNLI to identify people who are experiencing difficulties in participating in various valued life events; therefore, the sensitivity and specificity of using C-RNLI to identify frail older people who experience difficulties in participating in valued life events should be investigated based on the criteria suggested by Fairhall et al. [16].

## Conclusion

The RNLI has been widely used to measure participation restriction, but few attempts have been made to investigate its psychometric properties when used to measure participation restriction. The findings of this study demonstrate that this instrument is a reliable and valid tool for measuring participation restriction among pre-frail and frail older people. A CFA showed that the C-RNLI contains two factors, namely: participation in physical activities (items 1, 2, 4, and 5) and participation in social events (items 3 and 6–11). It is necessary to examine more psychometric properties of the C-RNLI in populations other than those suffering from frailty, since different target populations may have different patterns of participation restriction.

## Abbreviations

CGDS-SF: The Cantonese version of the Geriatric Depression Scale Short Form
C-RNLI: The Chinese version of the Reintegration to Normal Living Index
HKC-IADL: The Hong Kong Chinese version of the Lawton instrumental activities of daily living scale
